# On the Use of Phenolic Compounds Present in Citrus Fruits and Grapes as Natural Antioxidants for Thermo-Compressed Bio-Based High-Density Polyethylene Films

**DOI:** 10.3390/antiox10010014

**Published:** 2020-12-25

**Authors:** Sandra Rojas-Lema, Sergio Torres-Giner, Luis Quiles-Carrillo, Jaume Gomez-Caturla, Daniel Garcia-Garcia, Rafael Balart

**Affiliations:** 1 Technological Institute of Materials (ITM), Universitat Politècnica de València (UPV), Plaza Ferrándiz y Carbonell 1, 03801 Alcoy, Spain; luiquic1@epsa.upv.es (L.Q.-C.); jaugoca@epsa.upv.es (J.G.-C.); dagarga4@epsa.upv.es (D.G.-G.); rbalart@mcm.upv.es (R.B.); 2 Escuela Politécnica Nacional, Quito 170517, Ecuador; 3 Research Institute of Food Engineering for Development (IIAD), Universitat Politècnica de València (UPV), Camino de Vera s/n, 46022 Valencia, Spain

**Keywords:** green polyolefin, natural antioxidants, polyphenols, flavonoids, thermo-oxidative stability, active food packaging

## Abstract

This study originally explores the use of naringin (NAR), gallic acid (GA), caffeic acid (CA), and quercetin (QUER) as natural antioxidants for bio-based high-density polyethylene (bio-HDPE). These phenolic compounds are present in various citrus fruits and grapes and can remain in their leaves, peels, pulp, and seeds as by-products or wastes after juice processing. Each natural additive was first melt-mixed at 0.8 parts per hundred resin (phr) of bio-HDPE by extrusion and the resultant pellets were shaped into films by thermo-compression. Although all the phenolic compounds colored the bio-HDPE films, their contact transparency was still preserved. The chemical analyses confirmed the successful inclusion of the phenolic compounds in bio-HDPE, though their interaction with the green polyolefin matrix was low. The mechanical performance of the bio-HDPE films was nearly unaffected by the natural compounds, presenting in all cases a ductile behavior. Interestingly, the phenolic compounds successfully increased the thermo-oxidative stability of bio-HDPE, yielding GA and QUER the highest performance. In particular, using these phenolic compounds, the onset oxidation temperature (OOT) value was improved by 43 and 41.5 °C, respectively. Similarly, the oxidation induction time (OIT) value, determined in isothermal conditions at 210 °C, increased from 4.5 min to approximately 109 and 138 min. Furthermore, the onset degradation temperature in air of bio-HDPE, measured for the 5% of mass loss (T_5%_), was improved by up to 21 °C after the addition of NAR. Moreover, the GA- and CA-containing bio-HDPE films showed a high antioxidant activity in alcoholic solution due to their favored release capacity, which opens up novel opportunities in active food packaging. The improved antioxidant performance of these phenolic compounds was ascribed to the multiple presence of hydroxyl groups and aromatic heterocyclic rings that provide these molecules with the features to permit the delocalization and the scavenging of free radicals. Therefore, the here-tested phenolic compounds, in particular QUER, can represent a sustainable and cost-effective alternative of synthetic antioxidants in polymer and biopolymer formulations, for which safety and environmental issues have been raised over time.

## 1. Introduction

The packaging industry is currently searching for sustainable novel materials that can provide food quality and safety but are also able to successfully reduce the fossil fuel dependence of polymers and contamination of single-use plastics [1]. In the food packaging market, biopolymers represent the most relevant option to replace current petrochemical polymers [2]. Among biopolymers, bio-based high-density polyethylene (bio-HDPE) is well positioned in the today’s biopolymer industry. Bio-HDPE shows the sustainable benefit of being renewably produced at the industrial level by the addition polymerization of ethylene obtained by catalytic dehydration of bioethanol derived from sugar cane, from which it takes the name of “green” HDPE [3]. Moreover, bio-HDPE shows the same properties as its petrochemical counterpart, that is, high-density polyethylene (HDPE), which includes, for instance, high mechanical performance, water barrier, and chemical resistance [4,5].

Bio-HDPE can be manufactured into food packaging articles and food contact disposables by conventional melt-processing technologies such as injection molding and extrusion. However, it also degrades easily in oxidizing atmospheres during melt processing [6]. In particular, it can undergo thermal degradation between 160 and 200 °C [7,8]. Therefore, adequate stabilization by the addition of processing stabilizers, such as synthetic antioxidants, is essential to protect bio-HDPE during the manufacture of packaging articles and also hinders its degradation during use, which usually results in the gradual deterioration of its physical properties. The function of an antioxidant is to prevent the propagation steps of oxidation. Antioxidants can be divided into two main groups, depending on the method by which they prevent oxidation. Primary antioxidants (e.g., phenols, amines, or metal salts) act as radical scavengers and function by donating their reactive hydrogen to the peroxy free radical to avoid the propagation of subsequent free radicals. The antioxidant free radical is rendered stable by electron delocalization. Secondary antioxidants (e.g., organophosphites or thioesters) retard oxidation by preventing the proliferation of alkoxy and hydroxyl radicals by decomposing hydroperoxides to yield nonreactive products.

Nowadays, the plastic industry usually employs a synergistic combination of primary and secondary antioxidants to provide a higher stability to the polymer during melt processing but also to protect it from degradation during shelf life. Nevertheless, despite the fact that these additives can provide good protection from oxidation at a relatively low cost, there are some uncertainties regarding the effect of their reaction products on human health [9] and also the hazard they can produce to the environment in the long term [10]. Therefore, the use of natural additives has been currently envisioned as a safe and environmentally friendly alternative to replace synthetic ones. Natural phenolic compounds and other renewable substances with antioxidant activity can be found in vegetables and fruits and are known to have a beneficial effect on human health. Examples of these natural compounds include α-tocopherol (vitamin E), curcumin, or dihydromyricetin (DMH), which have been reported to stabilize polyolefins [10]. In this regard, some studies have already reported that the incorporation of natural antioxidants, for instance flavonoids, can reduce or avoid the degradation of polypropylene (PP) [11] and low-density polyethylene (LDPE) [12,13].

Natural antioxidants, in most cases, are found in vegetables and fruits, which contain lignin and polyphenols such as carotenes, flavonoids, or tannins [14,15]. From a more sustainable approach, natural antioxidants can also be obtained from agro-food wastes to promote the so-called Circular Bioeconomy concept [16]. In this context, citrus fruits and grapes contain a considerable amount of different phenolic compounds in leaves, peels, pulp, and seeds. Citrus peel is the primary waste product from juice processing, which represents an important inexpensive source of polyphenols, such as flavonoids and phenolic acids [17]. For instance, orange peels contain about 13.5 g of flavonoids per kg of dry matter, in which naringin (NAR) or 4′,5,7-trihydroxyflavanone 7-rhamnoglucoside, is among the most important ones [18]. NAR is a phenolic compound whose chemical structure is based on two aromatic rings connected by a bridge consisting of three carbon atoms, forming an oxygenated heterocycle [19,20]. Phenolic compounds are also present mainly in the skins and seeds of red grape berries. Gallic acid (GA) or 3,4,5-trihydroxybenzoic acid is another phenolic compound that is present in grapes and other fruits such as strawberries and bananas as well as in the leaves of several plants [21]. GA is a water soluble phenolic acid that has been widely explored for its antioxidant effects, but also as a homeopathic and anti-inflammatory drug or, more recently, as an anticarcinogenic agent, among other uses, in the medical and pharmaceutical fields [22]. Caffeic acid (CA), or 3,4-dihydroxybenzeneacrylic acid, is considered as the predominant phenolic acid contributing to the hydroxycinnamic acid content in coffee and various citric fruits. CA has a high antioxidant effect, performs as a cancer inhibitor, and shows some antimicrobial activity [23,24]. Finally, quercetin (QUER), or 3,3′,4′,5,6-pentahydroxyflavone, is another relevant metabolite flavonoid found in some plants and foods, such as onions, grapes, berries cherries, broccoli, and citrus fruits [25,26]. QUER shows both excellent antioxidant and anticancer activities [27].

This research study focuses, for the first time, on the use of the phenolic compounds NAR, GA, CA, and QUER present in citrus fruits and grapes as natural antioxidants for bio-HDPE films. To this end, the different natural compounds were melt-mixed with bio-HDPE at 0.8 parts per hundred resin (phr) and the resultant pellets were shaped into films by thermo-compression. In order to determine the best performing antioxidants and their potential applications in sustainable food packaging, the resultant thermo-compressed bio-HDPE films were characterized in terms of their optical properties and morphology, chemical characteristics, and mechanical and thermal properties as well as their antioxidant capacity.

## 2. Experimental

### 2.1. Materials

Bio-HDPE was provided by Braskem (São Paulo, Brazil) as SHA7260 and supplied in pellets form by FKuR Kunststoff GmbH (Willich, Germany). It has a density of 0.955 g/cm^3^ and its weight- and number-average-molecular weights (M_W_, M_n_) are 192,099 g/mol and 10,475 g/mol, which results in a dispersity (Ð) value of 18.3 [28]. This grade has been developed for injection molding and it shows a melt flow index (MFI) of 20 g/10 min (2.16 kg, 190 °C), while its minimum renewable content is 94% as determined by ASTM D6866.

**Figure 1:**
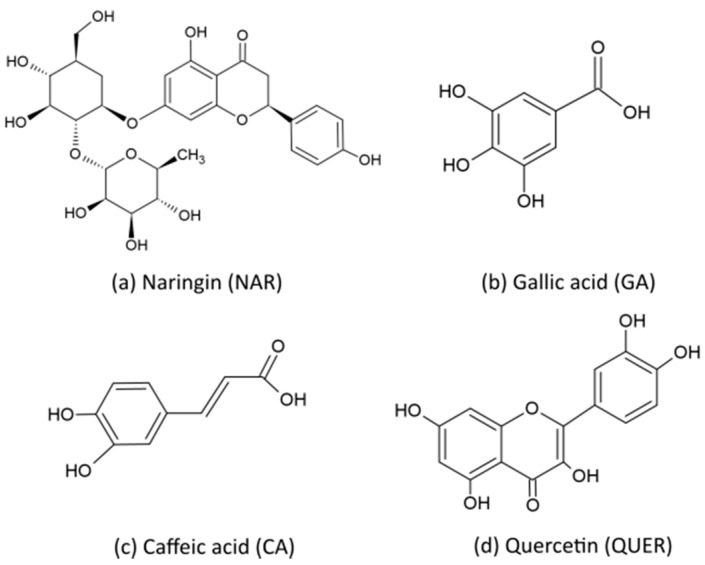
Chemical structure of (**a**) Naringin (NAR); (**b**) Gallic acid (GA); (**c**) Caffeic acid (CA); and (**d**) Quercetin (QUER).

Natural phenols were all supplied commercially, in powder form, by Sigma-Aldrich S.A. (Madrid, Spain). NAR, with CAS number 10236-47-2, and a product number N1376 is flavanone glycoside ≥ 90% purity and derived from citrus fruit, with a molecular weight (M_W_) of 580.53 g/mol. GA, with product number G7384, and a CAS number of 149-91-7, has a purity over 97.5% and a M_W_ of 170.12 g/mol. CA, with a CAS number 331-39-5, and a product number C0625, has a purity of ≥98.0% and M_W_ of 180.16 g/mol. QUER, with a CAS number of 117-39-5, purity of ≥95% and a M_W_ of 302.24 g/mol, was provided with the product number Q4951. The chemical structures of these phenolic compounds are gathered in Figure 1.

### 2.2. Manufacturing of Films

Bio-HDPE were manually premixed in a zipper bag with each natural antioxidant and then processed by melt compounding in a co-rotating twin-screw extruder (Construcciones Mecánicas Dupra, S.L., Alicante, Spain). The screw diameter is 25 cm and its length-to-diameter ratio (L/D) is 24. Further details about the extruder can be found elsewhere [29]. During melt processing, the temperature profile was set as follows: 140 °C (hopper)–150 °C–155 °C–160 °C (die), whereas screw rotation speed was adjusted to 20 rpm. The extruded strands were pelletized with an air-knife unit. The resultant pellets were dried at 60 °C for 72 h to remove moisture. Table 1 gathers the set of compositions prepared.

Bio-HDPE films were obtained by thermo-compression of the melt-compounded pellets using a 10-ton hydraulic press from Robima S.A. (Valencia, Spain) equipped with two hot aluminum plates and a temperature controller from Dupra S.A. (Castalla, Spain) [30]. The process was carried out at 130 °C, in which approximately 5 g of pellets were placed between the two hot plates and a pressure of 40 MPa was applied for 3 min. Films sizing 10 cm × 10 cm, with a mean thickness of approximately 250 μm, were attained. The films were stored at room conditions, that is, 23 °C and 50% relative humidity (HR), for at least 15 days in dark conditions before characterization.

### 2.3. Characterization of Films

#### 2.3.1. Color Measurements

A colorimetric spectrophotometer Konica CM-3600d Colorflex-DIFF2, from Hunter Associates Laboratory, Inc. (Reston, VA, USA), was used to determine the color coordinates and color changes of the films. Calibration of the instrument was performed with a white standard tile. The CIE Lab color space coordinates L*, a*, b* were determined using the standard illuminant D65 and an observer angle of 10°. In this system, L* represents luminance, where L* = 0 indicates dark and L* = 100 means lightness, whereas the color coordinates a* and b* represent color changes from red to green and from yellow to blue, respectively. The color difference (ΔE_ab_*) was calculated using Equation (1):

(1) $$\Delta E_{ab}{}^{*}=\sqrt{{\left(\Delta L^{*}\right)}^{2}+{\left(\Delta a^{*}\right)}^{2}+{\left(\Delta b^{*}\right)}^{2}}$$

where ΔL*, Δa*, and Δb* represent the variations in the L*, a*, and b* coordinates, respectively, between the neat bio-HDPE film and the films of bio-HDPE containing the natural antioxidants. The average values of at least 5 readings were reported. The color changes on the films were evaluated based on the ΔE_ab_* values according to the following assessment [31]: <1 means an unnoticeable color difference, values ranging between 1–2 indicate a slight difference in color that can only be noticed by an experienced observer, values in the 2–3.5 range indicate a noticeable difference by an inexperienced observer, values in the range of 3.5–5 indicate a clear noticeable difference, and values >5 indicate that different colors are noticeable.

#### 2.3.2. Microscopy

Morphologies of the fracture surfaces of the bio-HDPE films were observed by field emission scanning electron microscopy (FESEM). A ZEISS ULTRA 55 FESEM microscope (Oxfrod Instruments, Abingdon, UK) was used at an acceleration voltage of 2 kV. To this end, the film samples were cryo-fractured by immersion in liquid nitrogen and, thereafter, coated with a gold-palladium alloy (Quorun Technologies Ltd. EMITECH mod. SC7620 sputter coater, East Sussex, UK). 

#### 2.3.3. UV-Vis Spectroscopy

A Cary Series Ultraviolet-Visible-near-IR (UV–Vis–NIR) spectrophotometer (Agilent Technologies, Inc., Santa Clara, CA, USA) was used to obtain the UV–Vis absorption spectra of the bio-HDPE films. An incident radiation wavelength between 200 and 500 nm was set. All samples were studied in their thin film form and three replicates were tested.

#### 2.3.4. Infrared Spectroscopy

A chemical analysis of the as-received natural antioxidants and the bio-HDPE film samples was also performed by Attenuated Total Reflectance—Fourier Transform Infrared Spectroscopy (ATR-FTIR) spectroscopy. Tests were performed in a Pekin-Elmer equipment Spectrum BX FTIR (Beaconsfield, UK) coupled with an ATR MIRacle™ Pike Technologies (Madison, WI, USA). Twenty scans were averaged in the region from 4000 to 600 cm^−1^ at a resolution of 4 cm^−1^.

#### 2.3.5. Mechanical Tests

Tensile tests were carried out on rectangular bio-HDPE film samples with a total length and width of 100 mm and 10 mm, respectively. A universal test machine (Elib 50 S.A.E. Ibertest, Madrid, Spain) was used following the ISO 527-3:2018 guidelines. A load cell of 10 kN and a cross-head speed of 2 mm/min were used during the tests. Measurements were performed at room conditions and at least six films of each sample were analyzed.

#### 2.3.6. Thermal Tests

Differential scanning calorimetry (DSC) was performed on the bio-HDPE films using an average weight sample of 5–7 mg in a Mettler-Toledo 821 calorimeter (Mettler-Toledo, Schwerzenbach, Switzerland). The samples were placed in 40-μL aluminum-sealed crucibles and two types of DSC tests were performed. The first test was aimed to identify the onset oxidation temperature (OOT) and it was based on a dynamic heating ramp from 30 to 350 °C at a heating rate of 5 °C/min in air atmosphere. The second test, which allowed to obtain the oxidation induction time (OIT), consisted of a heating ramp from 30 to 210 °C at a heating rate of 5 °C/min in air atmosphere followed by an isotherm at 210 °C for a period of 300 min. The melting temperature (T_m_) was determined from the heating step during the first test. Additionally, the degree of crystallinity (*X_C_*) was calculated from the first heating ramp using Equation (2):

(2) $$X_{C}\left(\%\right)=\left[\frac{\Delta H_{m}-\Delta H_{CC}}{\Delta H_{m}^{0}\cdot \left(1-w\right)}\right]\cdot 100$$

where Δ*H_m_* (J·g^−1^) and Δ*H_CC_* (J·g^−1^) represent, respectively, the melt and cold crystallization enthalpies of bio-HDPE, while Δ*H_m_*^0^ (J·g^−1^) is the melt enthalpy of a theoretically fully crystalline bio-HDPE (293 J/g) [32] and the term (1 *− w)* represents the weight fraction of bio-HDPE in the sample.

Thermogravimetric analysis (TGA) was also conducted for the bio-HDPE film samples using an average weight of 15–25 mg in a PT1000 from Linseis (Selb, Germany). Samples were first placed in standard 70-μL alumina crucibles and subjected to a heating program in air atmosphere from 30 to 700 °C at a heating rate of 20 °C/min. The temperature measured for a mass loss of 5% (T_5%_) was considered as the onset degradation temperature, while the temperature at the maximum degradation rate (T_deg_) was determined from the first derivative thermogravimetry (DTG) curves. All the thermal tests were performed in triplicate.

#### 2.3.7. Antioxidant Measurements

The antioxidant activity of the films containing the phenolic compounds was analyzed by the inhibition assay of 2,2-diphenyl-1-picrylhydrazyl radical (DPPH), which is a stable and commercially available organic nitrogen radical that shows hydrogen acceptor ability towards antioxidants. In methanol solution, DPPH presents a strong violet color that fades as the reduction proceeds in the presence of an antioxidant substance and the progress of this reaction can be recorded by spectrophotometry [33]. The antioxidant activity of the bio-HDPE films was determined following the procedure described by Goñi et al. [34]. To this end, a stock solution of DPPH (Sigma Aldrich S.A) at 0.025 g/L in methanol (≥99.8%, HPLC grade, Panreac Quimica S.A., Barcelona, Spain) was prepared and placed into dark glass flasks. Thereafter, about 100 mg of each film sample was immersed in 5 mL of the stock solution in the flasks, which corresponds to a maximum concentration of antioxidant of nearly 160 ppm. The flaks were immediately kept hermetically closed and protected from light without stirring for one week. Absorbance measurements of the different samples were taken at 1, 24, 96, and 168 h. Vials without film sample were also prepared under the same conditions as the control. After each period, the films were removed from the vials and the absorbance of the resultant solution was measured using a Cary Series UV–Vis–NIR spectrophotometer at 517 nm, where the unpaired electron of the free DPPH radical presents maximum absorbance. The percentage of DPPH inhibition of each film was determined using Equation (3), which also takes into account the absorbance of the films in methanol without DPPH as blank [35]:

(3) $$DPPH\;Inhibition\;\left(\%\right)=\;\left[\frac{A_{Control}-\left(A_{sample}-A_{blank}\right)}{A_{control}}\right]\cdot 100$$

where $A_{control},\;A_{blank},\;\mathrm{and}\;A_{sample}$ correspond to the absorbance values of the DPPH solution without film, methanol with film, and the DPPH solution with film, respectively. Measurements were done in triplicate.

### 2.4. Statistical Analysis

Results were evaluated at 95% confidence level (*p* ≤ 0.05) by one-way analysis of variance (ANOVA) according to Tukey’s test for the significant differences among the samples with the use of OriginPro 8 software (OriginLab Corporation, Northampton, MA, USA).

## 3. Results and Discussion

### 3.1. Optical and Morphological Properties

**Figure 2:**
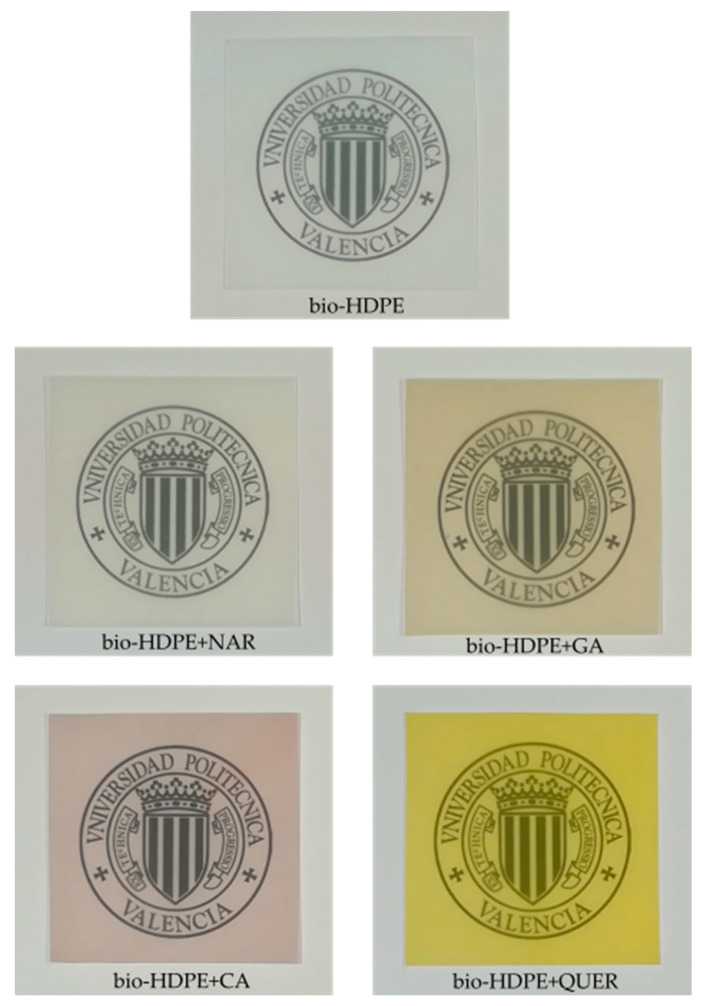
Visual appearance of the thermo-compressed bio-based high-density polyethylene (bio-HDPE) films containing naringin (NAR), gallic acid (GA), caffeic acid (CA), and quercetin (QUER).

In food packaging applications, color and transparency are important attributes of films. The visual aspect of the neat bio-HDPE film and the bio-HDPE films containing the phenolic compounds is shown in Figure 2. As it can be seen in the images, there was a clear variation in the film color after the incorporation of the different phenolic compounds. Whereas a low but still noticeable variation in color was produced in the NAR-containing bio-HDPE film, the other natural antioxidants yielded strong color changes in the films. The different colors attained in the bio-HDPE films can be ascribed to the natural and intrinsic color of each phenolic compound due to the most natural antioxidants having inherently strong colors [10]. The color of the antioxidants depends on different factors such as the number of hydroxyl (–OH) and methoxyl (–OCH_3_) groups as well as their position in the molecule [36]. For instance, the color tends to be blue if the chemical structure shows a large number of –OH groups, while the presence of –OCH_3_ groups favors the development of red tonalities [15]. As an example, the natural antioxidant curcumin is known to show a strong yellow color due to the chemical structure of this flavonol-type flavonoid [37]. In the case of QUER, its characteristic intense yellow color has been particularly ascribed to the conjugation of the double bond in ring C with the delocalized π electrons of ring B [38]. Furthermore, the brown color of GA can be attributable to chemical moieties and the presence of remaining pigments from fruits [39]. For CA, the formation of chlorogenic acid has been reported to produce certain brown discoloration in food [40]. When these natural antioxidants are mixed with biopolymers, as expected, the final samples tend to be colored [15]. For instance, Kirschweng et al. [38] reported that QUER colored polyolefins to yellowish red at a content as low as 5 ppm, while discoloration was intense at 500 ppm. However, the induced color slightly decreased during consecutive processing steps due to consumption of the flavonoid.

Color differences of the bio-HDPE films were quantified in Table 2 by the values of L* (luminance) and the a* (green to red) and b* (blue to yellow) coordinates as well as the color change, that is, ΔE_ab_*, after the incorporation of the different antioxidants. It can be noticed that the addition of 0.8 phr of each antioxidant caused a significant decrease in the luminance L* value, being more intense in the sample with CA, indicating that lightness was reduced in all the bio-HDPE films. One can observe that the GA-containing film developed higher values of a* and b* of 0.19 and 13.26, respectively, corroborating the reddish yellow coloring. Higher values of a*, that is, 4.80, but lower of b*, that is, −1.32, were attained for the bio-HDPE film with CA, as an indication of the developed blueish red hue. The highest color variation was observed for the addition of QUER, having values of 0.57 and 49.00 for a* and b*, respectively, and supporting the development of a strong yellow color in the biopolymer film sample. In all cases, the ΔE_ab_* values were higher than 5, indicating that different colors can be easily noticed by an unexperienced observer. The highest color change was produced after the addition of QUER, having a value of ΔE_ab_* of 54.47. Similarly, Marcos et al. [41] showed that the incorporation of 2.82 wt% of α–tocopherol and olive leaf extract with antioxidant properties into poly(butylene adipate-*co*-terephthalate) (PBAT) yielded ΔE_ab_* values of 9.04 and 40.40, respectively, indicating that natural antioxidants are very prone to coloring biopolymers. Furthermore, it is noteworthy to indicate that color intensities can be related to the different solubility of the natural additives in polyolefins [42]. It is also important to mention that, despite the differences in color, the contact transparency of the films was nearly unaffected.

**Figure 3:**
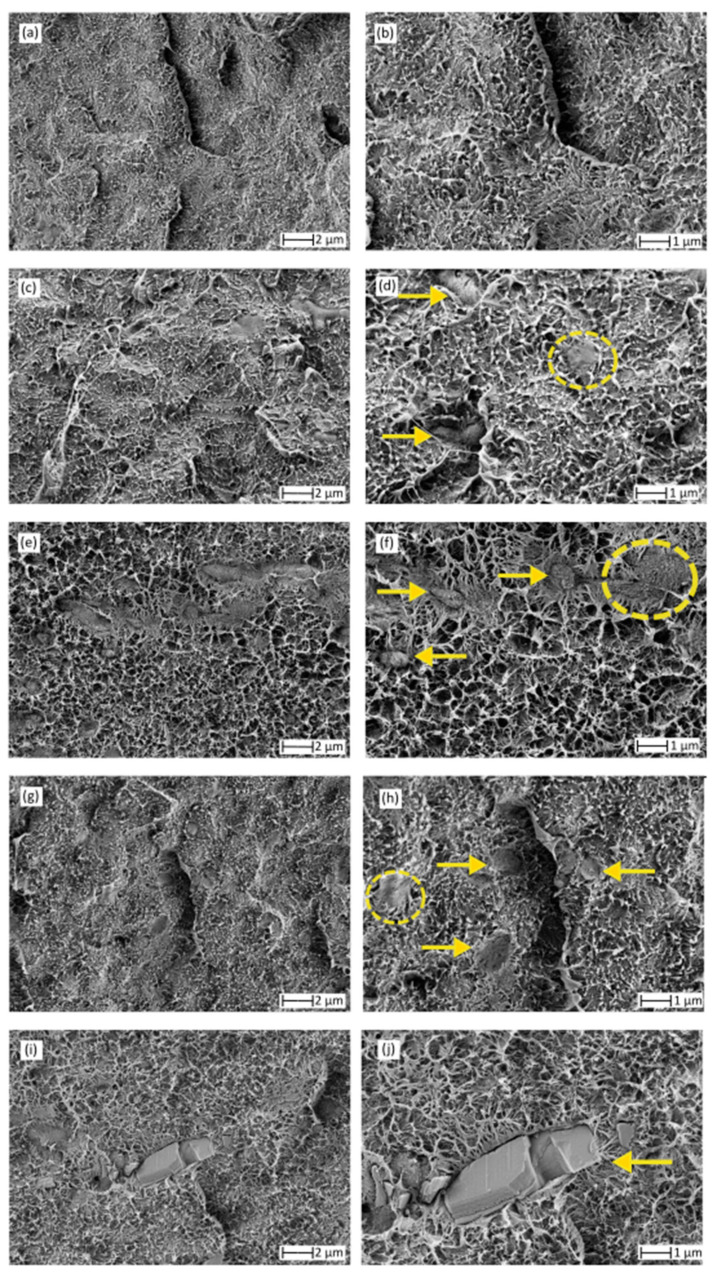
Field emission scanning electron microscopy (FESEM) micrographs of the cryo-fracture surfaces of the thermo-compressed films of: (**a**,**b**) neat bio-based high-density polyethylene (bio-HDPE); (**c**,**d**) bio-HDPE + naringin (NAR); (**e**,**f**) bio-HDPE + gallic acid (GA); (**g**,**h**) bio-HDPE + caffeic acid (CA); (**i**,**j**) bio-HDPE + quercetin (QUER). Left images were taken at 5000× with scale of 2 µm and right images were taken at 10,000× with scale of 1 µm. Yellow arrows indicate the presence of the phenolic compounds.

Figure 3 shows the FESEM images of the cryo-fracture surfaces of the film samples, in which the left images offers a general overview of the film surfaces after fracture while the right images gives more detail about the presence of the phenolic compounds. The latter FESEM micrographs were marked by yellow arrows and circles to indicate the presence of the natural compounds. In Figure 3a,b, one can see that the surface of bio-HDPE showed some macro-cracks and the presence of several filaments that correspond to the plastic deformation of the green polyolefin during fracture. This morphology is in agreement with previous works for HDPE [43,44] and it is an indication of the high ductility of the film sample. Figure 3c,d shows the cryo-fracture surface of the bio-HDPE film containing NAR, in which the same morphology can be seen with some round microparticles of the polyphenol embedded in the biopolymer matrix. A very similar fracture was attained during the morphological analysis of the cryo-fracture surface of the GA-containing bio-HDPE film, shown in Figure 3e,f, where round and well dispersed microparticles of the phenolic compound were observed. Indeed, the morphology of GA is typically characterized by crystals of a small size and a regular shape, with an apparently smooth surface [45]. In the case of the bio-HDPE film containing CA, shown in Figure 3g,h, some flakes of the natural compound were also embedded in the green polyolefin matrix. According to Luzi et al. [46], CA presents an irregular cubic crystal structure with a length distribution ranging between 5 and 50 μm. Finally, larger particles with a mineral-like aspect corresponding to QUER can be observed in Figure 3i,j. The latter morphology is in agreement with previous studies that have reported that this flavonoid, in its powdered form, consists of irregular rod structures [47,48]. Therefore, the FESEM analysis confirmed the effective dispersion of the natural antioxidants, though the morphology of the fracture surfaces of the bio-HDPE films was nearly unaffected by the presence of the phenolic compounds.

### 3.2. Chemical Properties

**Figure 4:**
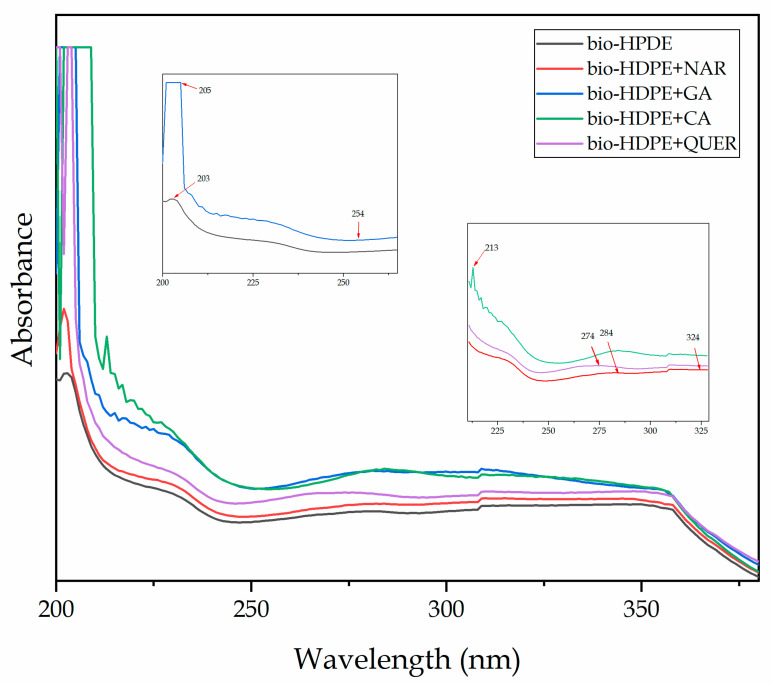
Ultraviolet–Visible (UV–Vis) spectra of the thermo-compressed bio-based high-density polyethylene (bio-HDPE) films containing naringin (NAR), gallic acid (GA), caffeic acid (CA), and quercetin (QUER).

UV–Vis spectroscopy was carried out on the compression-molded films to verify the presence of the phenolic compounds after their incorporation into the bio-HDPE matrix. Figure 4 shows the UV–Vis spectra obtained for the different thin film samples. According to Anna et al. [49], the peak observed at approximately 203 nm in the bio-HDPE spectrum corresponds to the characteristic peak for olefin-conjugated carbonyl groups. For the UV–Vis spectra of the bio-HDPE films containing the natural antioxidants, major changes were observed in the band at approximately 205 nm, which can be related to the π → π* transitions within the aromatic ring of the phenolic molecules. For the NAR-containing bio-HDPE sample, the flavanone glycoside also showed the characteristic contributions for ring 1 and 2 structures, which were seen as low-intense and flat peaks at 284 and 324 nm, respectively [50,51]. In the spectrum of the bio-HDPE sample containing GA, in addition to the strong peak at 205 nm, showing oversaturation, a new peak appeared at 254 nm that is also due to the aromatic ring of the phenolic acid [52]. For the sample of bio-HDPE with CA, the characteristic peak in the UV-vis region of aromatic rings of phenols also produced a new band at 213 nm. The band at the shorter wavelength is known as the B-band and the one at longer wavelength as the C-band [53]. Finally, in the bio-HDPE film with QUER, one can observe the presence of a low-intense peak at 274 nm. According to Dolatabadi et al. [54], QUER, like most flavones and flavonols, exhibits two major absorption bands in the UV–vis region, one at 372 nm (band I), representing B-ring absorption of the cinnamoyl system, and the other at 256 nm (band II), which is considered to be associated with the absorption involving the A ring benzoyl system. Therefore, the presence of these new bands in the bio-HDPE films confirms the presence of the natural phenolic compounds in the green polyolefin.

**Figure 5:**
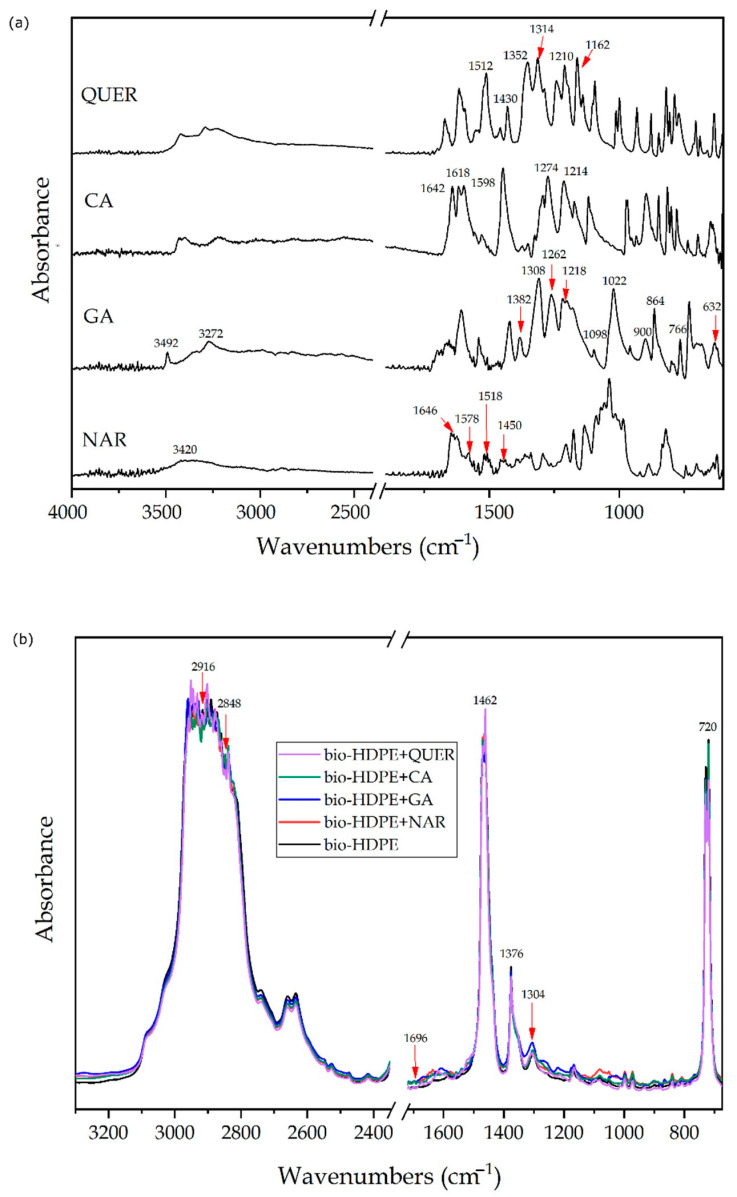
Fourier transform infrared (FTIR) spectra of: (**a**) natural antioxidants in powder form of, from bottom to top, naringin (NAR), gallic acid (GA), caffeic acid (CA), and quercetin (QUER); (**b**) thermo-compressed bio-based high-density polyethylene (bio-HDPE) films containing NAR, GA, CA, and QUER.

Chemical analysis of the natural antioxidants and their interaction with the bio-HDPE matrix was also carried out by FTIR spectroscopy. Figure 5 shows the ATR-FTIR spectra of the neat phenolic compounds in their power form and the bio-HDPE films after their incorporation. In Figure 5a, the spectra of the natural antioxidants are gathered, including arrows to show the main and relevant peaks. Regarding the NAR spectrum, one can observe the main characteristic peaks of –OH at 3420 cm^−1^ and for the C=O and C–O–C bonds at 1646 cm^−1^ [55]. The spectrum also showed the C–C signals that arise from the benzene ring at approximately 1578, 1518, and 1450 cm^−1^. In the case of GA, the peaks at 3492 and 3272 cm^−1^ correspond to the stretching modes of O–H, whereas the intense bands in the region from 1382 to 1060 cm^−1^ are ascribed to stretching and bending vibrations of C–C and C–H bonds of the aromatic ring, respectively. Additionally, in the region from 1308 to 1174 cm^−1^, different peaks related to the bending vibrations of C–H in the aromatic ring and O–H of the phenol group were observed [56]. In addition, the stretching and bending vibrations of C–O groups appeared in the region from 1021 to 628 cm^−1^. In the FTIR spectrum of CA, this phenolic acid was mostly characterized by its hydroxyl and carbonyl functional groups. One can observe the presence of two parallel peaks that correspond to the vibration of O–H group attached to the benzene ring centered at 1274 and 1214 cm^−1^ [57]. Furthermore, in the region from 1700 to 1600 cm^−1^, the broad band with three intensities at 1642, 1618, and 1598 cm^−1^ was due to C=O stretching. In this regard, Gunasekaran et al. [58] indicated that this signal habitually generates a strong band with high intensity and a relatively interference-free region. Finally, the main peaks seen for QUER were located from 1600 to 1100 cm^−1^, corresponding to the aromatic bending and stretching of C–C bonds and also O–H phenolic bending [59,60]. The most intense peaks were seen at 1512, 1430, 1352, 1314, 1210, and 1162 cm^−1^.

In order to analyze and confirm the presence of the phenolic compounds and ascertain their chemical interaction with the green polyolefin, FTIR spectroscopy was also performed on the thermo-compressed film samples. Figure 5b shows the ATR-FTIR spectra of neat bio-HDPE film and the bio-HDPE films containing the antioxidants. In the neat bio-HDPE spectrum, one can notice the presence of strong peaks centered at approximately 2916, 2848, 1462, and 720 cm^−1^. The first two peaks have been assigned to the asymmetric and symmetric stretching C–H of CH_2_, respectively, while the two other bands are due to bending and rocking deformations [61]. Other, less intense peaks, but still noticeable, were seen at 1376 and 1304 cm^−1^, corresponding to the CH_3_ symmetric and twisting deformations, respectively. A very weak peak was also observed at 1696 cm^−1^, which has been ascribed to carbonyl compounds formed as the oxidation products of polyethylene [62]. One can observe that the addition of the different natural antioxidants yielded small and very subtle variations in the peaks of the FTIR spectrum of bio-HDPE, indicating that their interaction with the green polyolefin was low. Some of the C–C and C–H signals, which arise from the benzene rings of the phenol groups in the 3000–2800 cm^−1^ region, altered some of CH_2_-related peaks present in bio-HDPE, particularly in the case of QUER-containing film. Similarly, other slight band changes were observed at lower wavenumbers, from 1400 to 1060 cm^−1^, which can be ascribed to stretching and bending vibrations of C–C and C–H bonds of the aromatic rings in the phenolic compounds. The band at 720 cm^−1^, which is due to rocking deformations of C–H bonds, shifted to lower wavenumbers after the addition of the phenolic compounds, this change being more significantly in the case of CA. It is also worth noting that the C=O stretching band, seen at approximately 1642 and 1618 cm^−1^, increased due to the presence of the natural antioxidants.

### 3.3. Mechanical Properties

**Figure 6:**
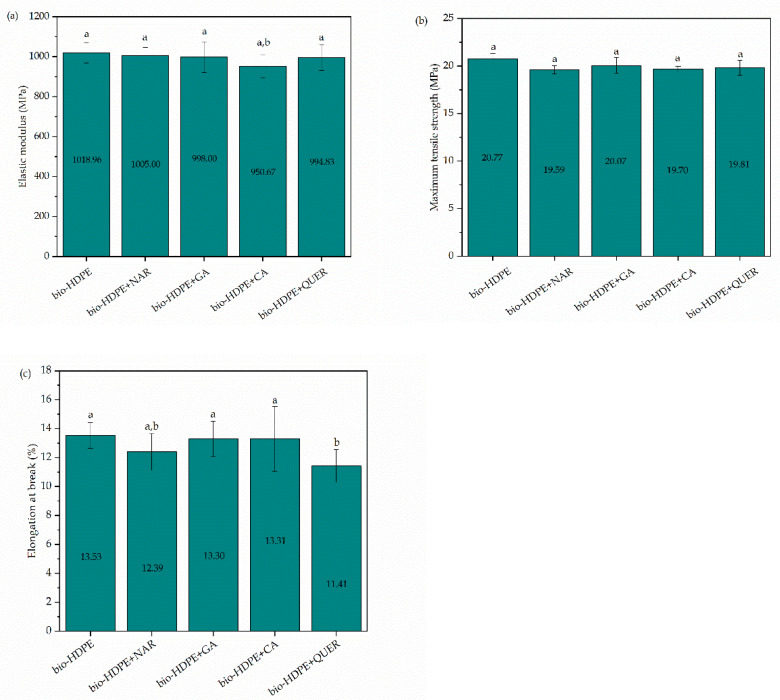
Mechanical properties of the thermo-compressed bio-based high-density polyethylene (bio-HDPE) films containing naringin (NAR), gallic acid (GA), caffeic acid (CA), and quercetin (QUER) in terms of: (**a**) elastic modulus; (**b**) maximum tensile strength; (**c**) elongation at break. ^a,b^ Different letters in the same property indicate a significant difference among the samples (*p* < 0.05).

Figure 6 shows bar graphs with the mechanical properties of the bio-HDPE films obtained after the tensile tests. Figure 6a–c respectively show the values of elastic modulus, maximum tensile strength, and elongation at break. In the case of the neat bio-HDPE, the film samples showed values of 1018.96 MPa, 20.77 MPa, and 13.53%, respectively. These mechanical properties indicate that the bio-HDPE film is an elastic and ductile material, which is in agreement with the cryo-fracture surfaces shown above and also in our previous study [63]. Results indicated that the addition of 0.8 phr of the different natural antioxidants did not significantly affect the mechanical properties, which also agrees with the previous morphological analysis. Despite that, it was observed that there was a slight decrease in the mechanical performance, in particular for the elongation-at-break values. The slight reduction in mechanical strength can be explained by the plasticizing effect caused by the addition of the phenolic compounds and also a decrease in the biopolymer’s crystallinity. In terms of ductility, however, the lower values can be related to the presence of phenol particles that were not soluble or showed low interaction with the bio-HDPE matrix, which is supported both by the cryo-fracture surfaces and the FTIR analysis of the films reported above. In any case, the attained differences were not significant since the content of additive was very low. The present results are also in agreement with previous studies reporting the mechanical properties of films containing natural antioxidants. For instance, Luzi et al. [64] showed that the incorporation of 5 wt% GA and umbelliferone yielded a slight decrease, but still not significant, in the overall mechanical performance of poly(ethylene-*co*-vinyl alcohol) (EVOH) films. Similarly, Sun et al. [65] showed that the addition of GA at 0.5 phr in chitosan films produced a slight decrease in their ductility. In the study performed by Colín-Chávez et al. [66], the addition of 2.9 wt% of marigold extract reduced the elongation-at-break values but increased the tensile strength of LDPE films. However, in all cases, no significant differences were observed. It is also worth mentioning the results agree with those obtained by Ramos et al. [67], after the incorporation of an equimolar mixture of carvacrol and thymol at 4, 6, and 8 wt% in PP films.

### 3.4. Thermal Properties

**Figure 7:**
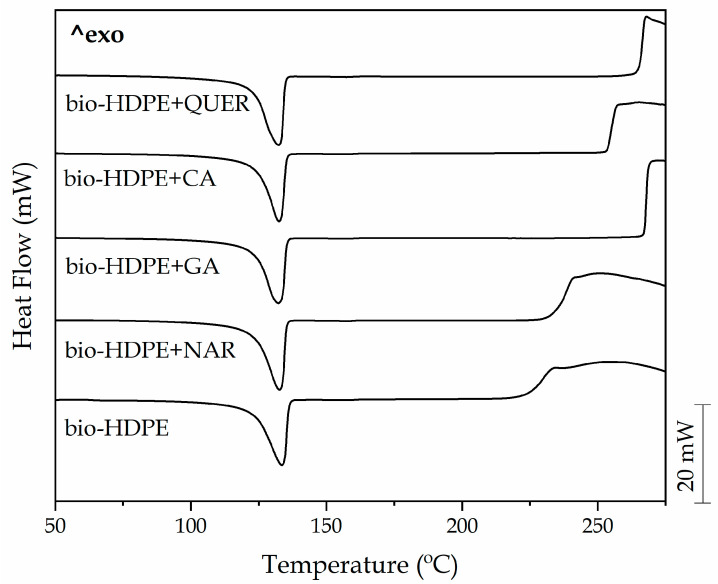
Heating curves obtained by differential scanning calorimetry (DSC) of the thermo-compressed bio-based high-density polyethylene (bio-HDPE) films containing naringin (NAR), gallic acid (GA), caffeic acid (CA), and quercetin (QUER).

The analysis of the thermal properties of the bio-HDPE films containing the different phenolic compounds was carried out by DSC and TGA. Figure 7 shows the dynamical DSC curves of the films, whereas Table 3 gathers the main thermal parameters obtained from the curves. One can notice that both the neat bio-HDPE film and the bio-HDPE films containing the natural antioxidant films showed a single sharp endothermic peak corresponding to the melting process of the green polyolefin crystals in the thermal range from 125 to 140 °C. In the case of the neat bio-HDPE film, this peak was centered at approximately 135 °C, which corresponds to the T_m_ value of bio-HDPE and is also in agreement with previous studies for this biopolymer [68]. The incorporation of the phenolic compounds reduced the T_m_ values by up to 3 °C, suggesting that the natural antioxidants restricted the development of the green polyolefin crystals. As a result, biopolymer crystals with lower lamellae thicknesses or more imperfections were developed after the incorporation of the natural antioxidants. This fact was further supported by a slight reduction of the crystallinity degree, that is, X_C_, from 64.1% for the neat bio-HDPE to values in the 58.5–63.9% range. However, this reduction was only significant in the case of the bio-HDPE films with CA. It is also worth indicating that, in all cases, high degrees of crystallinity were attained since the bio-HDPE grade used to produce the films was specifically designed for injection molding to develop thin-walled parts. Therefore, its high MFI, which is related to its low M_W_, could favor crystallization during film formation by thermo-compression. In this context, López-de-Dicastillo et al. [69] indicated that natural antioxidants, for instance ascorbic acid, ferulic acid, QUER or green tea extract, can induce a lower and more deficient crystallinity structure in EVOH. The fact that different levels of crystallinity were attained for each type of antioxidant could be related to the different morphologies and the role of the phenolic compounds as nucleating agents in bio-HDPE. In this sense, it has been reported that the presence of the antioxidant particles can produce two antagonistic effects, that is, a nucleating effect on the polyolefin, which induces the growth of a large number of crystals, but also a decrease in crystal size because of imperfections [70].

Table 3 also shows the T_onset_ of degradation, also called OOT, when it takes place in air or in any atmosphere rich in oxygen. This thermal oxidation can be seen in the DSC curves as the exothermic peak formed when high temperatures are reached. This parameter is considered as a common indicator used for ascertaining the polymer thermal-oxidative stability so it can be used to determine the thermal protection versus oxidation offered by added antioxidants [71]. One can observe that the neat bio-HDPE film started thermal oxidation at 223.7 °C, which is very similar to the value reported in our previous work for this green polyolefin [63]. It can also be observed that the different phenolic compounds provided different improvements in the thermal-oxidative stability of bio-HDPE. For instance, the addition of NAR resulted in an improvement in the OOT value of 8.5 °C, while CA yielded a more notable increase of 29.9 °C. The highest enhancement was observed, however, in the GA- and QUER-containing bio-HDPE films, which showed OOT values of 266.7 and 265.2 °C, respectively, providing an increase of 43 and 41.5 °C. Therefore, the incorporation of these natural phenols, particularly GA and QUER, greatly improved the thermal stability of the green polyolefin against oxidation at high processing temperatures.

In this context, phenols can prevent oxidation by neutralizing peroxide radicals. According to Kriston et al. [72], phenolic compounds can protect polyolefins from cross-linking reactions due to the formation of hydroperoxides by the transfer of a hydrogen atom from the phenolic fraction to the peroxy radical. Other authors such as Dopico-García et al. [73] also demonstrated that the use of natural extracts can successfully provide polyolefins with stabilization against degradation by thermal oxidation. Their antioxidant activity is based on the *o*-dihydroxy structure of catechin’s B ring, which confers greater stability to the radical form and participates in the delocalization of the electrons for the effective elimination of radicals. Moreover, different research groups have analyzed the effectiveness of some specific flavonoids as thermal stabilizers for polymer processing [74]. In particular, Zaharescu et al. [75] obtained an improvement in the thermal stability of ethylene-propylene-diene (EPDM) after the incorporation of NAR and CA. Both natural antioxidants delayed thermal oxidation significantly, especially when using large amounts of antioxidants. Similar improvements were obtained in the study performed by Samper et al. [11], in which 0.5 wt% of silibinin (SIL) and QUER acted as effective oxidative retardants for PP, successfully delaying T_onset_. In particular, QUER has shown to be an efficient antioxidant in LDPE and other polymers [42]. The high antioxidant capacity of QUER is based on the particular chemical structure of this flavonol. When it reacts with a free radical, it donates a proton and becomes a radical itself, but the resulting unpaired electron is delocalized by resonance in the benzene structure, making the QUER radical too low in energy to be reactive [76]. Furthermore, the B ring *o*-dihydroxyl groups, the 4-oxo group in conjugation with the 2,3-alkene, and the 3- and 5-hydroxyl groups, can also donate electrons to the rings and, thus, increase the number of available resonance forms. In the case of GA, the higher improvement attained can also be related to an improved dispersion due to its lower M_W_, generating a better contact with peroxyl radical in the biopolymer chains. For instance, contents as low as 0.3% of GA have successfully delayed the OOT value of bio-HDPE by more than 35 °C [63].

**Figure 8:**
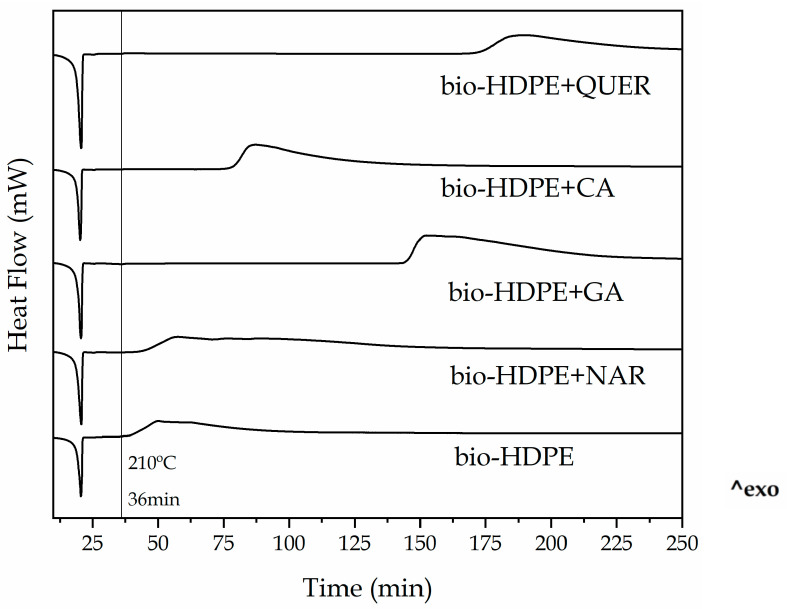
Isothermal curves obtained by differential scanning calorimetry (DSC) of the thermo-compressed bio-based high-density polyethylene (bio-HDPE) films containing naringin (NAR), gallic acid (GA), caffeic acid (CA), and quercetin (QUER).

The stabilizing effect of the natural antioxidants was also analyzed through OIT measurements. Figure 8 shows the isothermal curves of the bio-HDPE films when heated at 210 °C for a span time of 300 min. During the heating ramp, all the bio-HDPE samples first melted and then reached the selected temperature after 36 min. Thereafter, thermal oxidation was seen as an exothermic peak in the DSC curves and the OIT values were determined as the time taken to start thermal decomposition. This value can be very valuable to understand the degradation occurring in the green polyolefin when it is extruded or processed at high temperatures. As also shown in Table 3, one can see that neat bio-HDPE degraded in only 4.5 min after reaching 210 °C. Interestingly, in all cases, the addition of the different natural antioxidants successfully delayed the onset of thermal degradation. NAR, with an OIT of 9.2 min, enhanced the oxidative thermal exposure time of bio-HDPE by more than 100% though it provided the lowest improvement, as similar to that found above for OOT. The incorporation of CA, GA, and, more importantly, QUER achieved OIT values for bio-HDPE of approximately 42.7, 109.3, and 137.9 min, respectively. The here-attained OIT values point out that these natural phenolic compounds are very promising additives to improve the oxidative thermal degradation of bio-HDPE. Similar results have been reported for α-tocopherol, which was also compared in terms of thermal protection with synthetic antioxidants such as butylated hydroxytoluene (BHT) [77]. Similarly, the work of Li et al. [78] showed that the incorporation of 0.1 wt% of dendritic antioxidant delayed the OIT of PP and LDPE at 200 °C by approximately 40 and 50 min, respectively. Authors also demonstrated that the observed OIT values were higher than those obtained with commercial antioxidants Irganox^®^ 1010 and 3114 by BASF (Ludwigshafen, Germany), which are sterically hindered phenolic antioxidants and achieved values in the range of 15–25 min. However, it is worth mentioning that, in comparison with the present study, the quantities of antioxidant added was much lower. Other studies have reported the addition of higher contents of commercial antioxidants, such as Irganox^®^ L135 and L57 (BASF) at 0.5 wt%, a phenolic and amine antioxidants, respectively, showing OIT values of 3.58 min and 5.92 min for a lubricant base oil [79].

**Figure 9:**
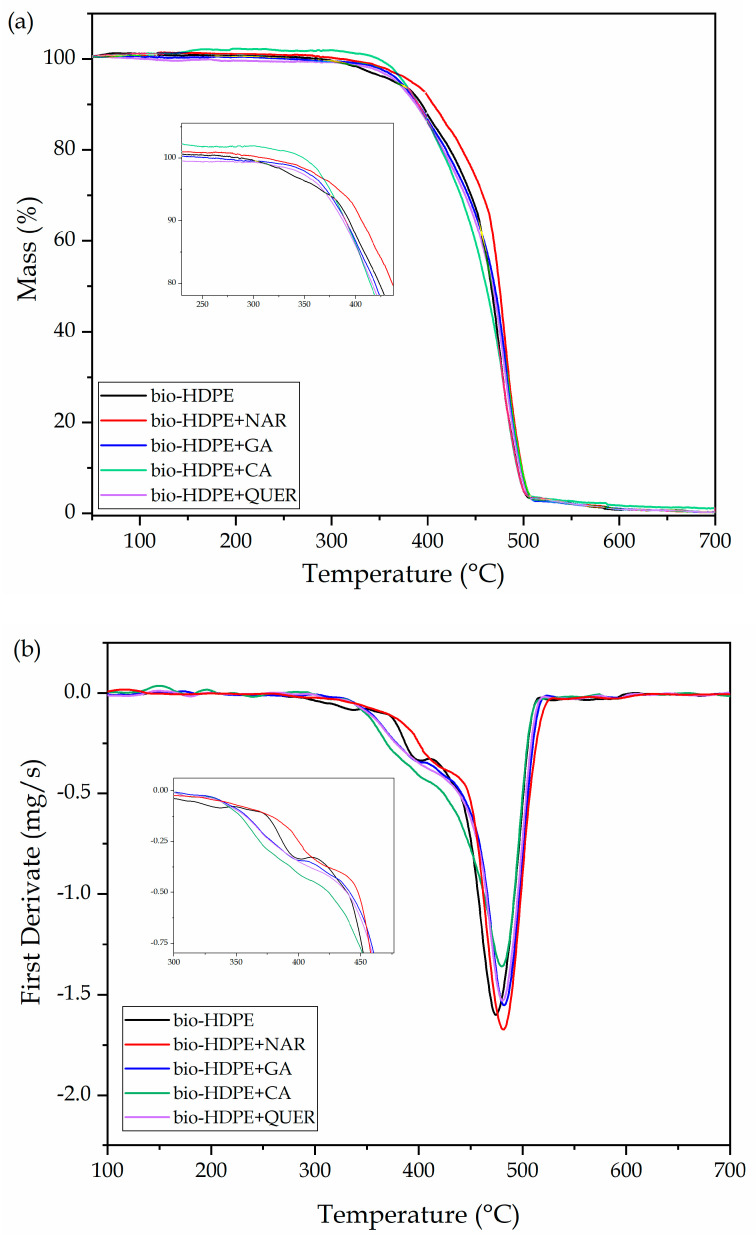
(**a**) Thermogravimetric analysis (TGA) and (**b**) first derivative (DTG) curves of the thermo-compressed bio-based high-density polyethylene (bio-HDPE) films containing naringin (NAR), gallic acid (GA), caffeic acid (CA), and quercetin (QUER).

TGA was also performed on the green polyolefin films to study the thermal stability of the samples after the incorporation of the different natural antioxidants. Figure 9 shows the TGA and DTG curves of the neat bio-HDPE film and the films of bio-HDPE with the phenolic compounds. The thermal values obtained from the TGA curves are summarized in Table 4. In Figure 9a, the evolution of mass (%) as a function of temperature is represented, while Figure 9b shows their DTG curves (mg/s). It can be observed that the neat bio-HDPE showed values of T_5%_ and T_deg_ of 355.1 and 473.7 °C, respectively. The addition of the natural antioxidants significantly delayed the onset of thermal degradation, that is, T_5%_, by 7–21 °C. Although a similar improvement was achieved in all cases, the highest T_5%_ value was attained for the NAR-containing bio-HDPE sample, showing a value of 376.1 °C. In the case of T_deg_, all the natural antioxidants yielded a slight improvement, of approximately 5–8 °C, indicating that their major contribution takes place during the initiation of thermal degradation. It is also worth indicating that, in all samples, thermal degradation of bio-HDPE occurred in a single step, as reported earlier [68]. However, it is possible to observe a lower thermal decomposition rate of bio-HDPE up to approximately 400 °C, which can be seen as a shoulder in the DTG curve, followed by a faster degradation rate. This phenomenon has been ascribed to two consecutive degradation steps in polyolefins, first the decomposition of the C–C covalent bonds by free radicals and then the sequential thermal degradation and breakdown of the polymer chains [80]. One can notice that this shoulder nearly vanished in the bio-HDPE films containing the phenolic compounds, suggesting that they actively participated as free radical scavengers due to the high reactivity of their hydroxyl substituents [63]. Finally, with regard to the residual mass, one can observe that all bio-HDPE films yielded very similar values from 0.25 to 0.30% at 700 °C.

The improvement in thermal stability reported by TGA also correlates well with the results obtained by other previous studies. For instance, Luzi, et al. [64] showed that 5 wt% of GA led to an increase in T_onset_ and T_deg_ of approximately 44 and 20 °C, respectively. The study performed by Samper et al. [11] indicated that the addition of QUER at 0.75 wt % in PP allowed to delay T_onset_ by nearly 32 °C, while NAR yielded a thermal improvement of 10 °C. Additionally, in the study carried out by Hernández-Fernández et al. [81], an improvement of approximately 30 °C in the T_onset_ of PP films after the addition of 0.1 wt% of CA was reported.

### 3.5. Antioxidant Activity

**Figure 10:**
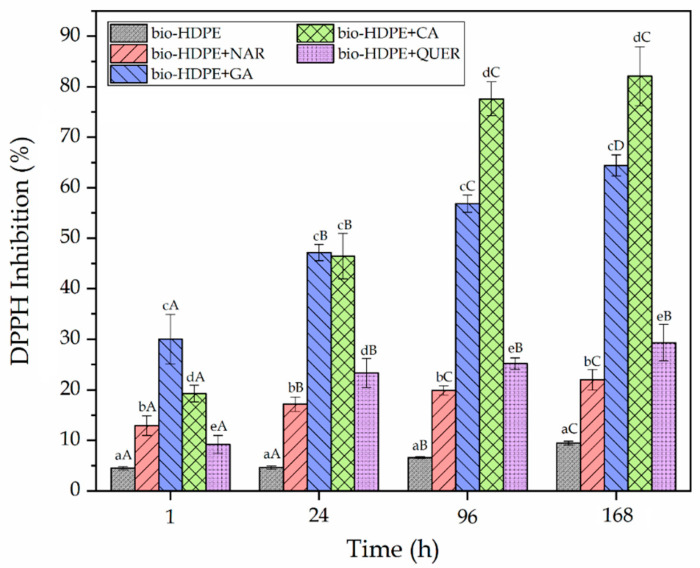
Percentage of 2,2-diphenyl-1-picrylhydrazyl radical (DPPH) inhibition of the thermo-compressed bio-based high-density polyethylene (bio-HDPE) films containing naringin (NAR), gallic acid (GA), caffeic acid (CA), and quercetin (QUER). ^a–e^ Different letters in the same period for different samples indicate a significant difference (*p* < 0.05). ^A–D^ Different letters for the same sample in different periods indicate a significant difference (*p* < 0.05).

Although the main objective of the present study was to improve the thermal oxidation stability of bio-HDPE by the addition of the natural phenolic compounds, the antioxidant activity of the films was also determined by a release mechanism. To this end, the DPPH free radical method was carried out immersing the films in methanol. This antioxidant assay is based on an electron-transfer that produces a color change in the alcoholic solution from violet/purple of the stable radical DPPH to the yellow colored diphenyl-picrylhydrazine, which can be followed by UV-Vis spectroscopy. Figure 10 shows the DDPH inhibition percentages for each sample at different times for a whole period of one week. It can be observed that all the phenolic compounds successfully provided antioxidant activity. Furthermore, the absorbances of all the DPPH solutions were nearly stabilized in 96 h, in most cases showing no significant differences, though the values slightly increased. The resultant antioxidant activity of the bio-HDPE films containing the natural compounds can be ascribed to their phenolic groups (ArOH), which could stabilize the DPPH radical (DPPH^•^) to its non-radical form (DPPH–H). This process has been reported to occur via two different mechanisms [82]: Equation (4a) a direct abstraction of phenol H-atom and Equation (4b) an electron transfer process from ArOH or its phenoxide anion (ArO−) to DPPH^•^, which can be summarized according to the proposed scheme:

(4a) ArOH + DPPH^•^ → ArO^•^ + DPPH–H

(4b) ArO^•^+ DPPH^•^ → products

The HAT mechanism Equation (4a) is predominant in apolar solvents, but in polar solvents, such as methanol, the ET mechanism Equation (4b) also becomes important due to it is capability of forming strong hydrogen bonds with the ArOH molecules [83]. One can also notice that the GA and CA antioxidants were shown to be 2–3 fold more active than NAR and QUER. In particular, whereas NAR and QUER reached a DPPH inhibition of up to 21.95% and 29.33%, respectively, GA and CA presented values of 64.42% and 82.09%. The differences attained among the bio-HDPE film samples can be mainly related to the different released amounts of each phenolic compound into the methanol medium. In the cases of GA and CA, their lower M_W_ values could favor their diffusion from the bio-HDPE matrix. The highest antioxidant activity attained for CA can be related to the fact that cinnamic acid derivatives are known to be more potent free radical scavengers than benzoic acid derivatives, such as GA, which is based on their enhanced resonance stabilization that arises from the conjugation of π electrons in the ring with the π bond in the side-chain [84]. It is also interesting to note that the neat bio-HDPE film showed a slight value of DPPH inhibition (9.45%). This result may be due to some radical scavenging capacity of the bio-HDPE itself and/or the presence of antioxidant added by the manufacturer. A similar effect was previously observed for linear low-density polyethylene (LLDPE), which was ascribed to a slow diffusion of DPPH from the methanol solution into the polyolefin film, decreasing its concentration [34]. From the above, the here-developed bio-HDPE containing the natural compounds can also be of interest for active food packaging, particularly in the case of GA and CA. For instance, the resultant films can avoid or delay biochemical reactions such as oxidation of fats and sugars caused by light that generates unpleasant aromas and flavors [85].

## 4. Conclusions

The present study has evaluated the potential use of different phenolic compounds present in citrus fruits and grapes, which can be found as food processing by-products of the juice industries, as natural antioxidants in bio-HDPE films. Results showed that the incorporation of NAR, GA, CA, and QUER at 0.8 phr into bio-HDPE was successfully achieved. In terms of optical properties, the natural compounds yielded significant colors to the green polyolefin, however the films were still contact transparent. The cryo-fracture surfaces indicated that the phenolic compounds were embedded and well dispersed in the bio-HDPE matrix and the fracture behavior remained unaltered. The chemical analyses confirmed the successful inclusion of the phenolic compounds in the bio-HDPE matrix, though their interaction with the green polyolefin matrix was low. The bio-HDPE films were very ductile and, although the natural antioxidants slightly reduced the mechanical properties due to their limited solubility in the green polyolefin matrix, differences were not significant due to the low content of natural additive incorporated. Regarding the resistance to thermal oxidation, the use of the phenolic compounds yielded very promising results. All the here-tested phenolic compounds successfully increased the oxidative thermal stability of bio-HDPE, showing GA and particularly QUER the highest performance. Finally, the antioxidant activity of the phenol-containing bio-HDPE films was assessed by the DPPH free radical method in methanol solution. The results showed that the GA- and CA-containing films presented high antioxidant properties and could be potential candidates for active food packaging applications. The improved antioxidant performance of GA, CA, and, more importantly, QUER, was ascribed to the presence of multiple hydroxyl groups and aromatic heterocyclic rings that provide these molecules with the features to permit the delocalization and the scavenging of free radicals.

Therefore, one can consider that the present phenolic compounds, which are naturally present in citrus fruits and grapes, can represent a suitable alternative to synthetic antioxidants, for which safety and environmental issues have been raised over time. In particular, QUER, a major representative of the flavonol subclass, have been shown to effectively improve the thermo-oxidative degradation of green polyolefins, which can allow the enlargement of their processing and applications in the packaging sector. Alternatively, the use of GA, and particularly CA, is very promising to develop active systems in food packaging applications by a release mechanism. Future works will explore the use of minimally processed phenolic extracts obtained from by-products of citrus and grape juices and related food industries. These phenolic compounds, which are contained mainly in the skins, albedos, and seeds of fruits and berries, can be released by hydrolysis and heat breakdown from certain esters or more complex molecules. The alternative use of the natural extracts will avoid the implementation of preparative chromatographic methods to isolate the pure phenolic compounds that, otherwise, could increase the cost and thus the viability of the valorization process. Furthermore, although these compounds are natural and show a great deal of potential for food packaging, their applications should also consider their possible migration and toxicological effects as well as negative effects on the sensory attributes, especially in terms of flavor.

## Figures and Tables

**Table 1:** Summary of compositions according to the weight (wt%) of bio-based high-density polyethylene (bio-HDPE) in which naringin (NAR), gallic acid (GA), caffeic acid (CA), and quercetin (QUER) were added as parts per hundred resin (phr) of bio-HDPE.

Sample	Bio-HDPE (wt%)	NAR (phr)	GA (phr)	CA (phr)	QUER (phr)
Bio-HDPE	100	0	0	0	0
Bio-HDPE + NAR	100	0.8	0	0	0
Bio-HDPE + GA	100	0	0.8	0	0
Bio-HDPE + CA	100	0	0	0.8	0
Bio-HDPE + QUER	100	0	0	0	0.8

**Table 2:** Color parameters (L*, a*, b*) and difference (ΔE_ab_*) of the thermo-compressed bio-based high-density polyethylene (bio-HDPE) films containing naringin (NAR), gallic acid (GA), caffeic acid (CA), and quercetin (QUER).

Film	L*	a*	b*	ΔE_ab_*
bio-HDPE	81.60 ± 0.52 ^a^	−1.82 ± 0.04 ^a^	−4.16 ± 0.11 ^a^	-
bio-HDPE + NAR	79.24 ± 0.26 ^b^	−3.53 ± 0.06 ^b^	5.11 ± 0.13 ^b^	9.73 ± 0.14 ^a^
bio-HDPE + GA	63.16 ± 0.29 ^c^	0.19 ± 0.10 ^c^	13.26 ± 0.31 ^c^	25.45 ± 0.41 ^b^
bio-HDPE + CA	55.48 ± 0.23 ^d^	4.80 ± 0.15 ^d^	−1.32 ± 0.11 ^d^	27.10 ± 0.22 ^c^
bio-HDPE + QUER	69.95 ± 0.49 ^e^	0.57 ± 0.25 ^e^	49.00 ± 1.49 ^e^	54.47 ± 1.54 ^d^

^a–e^ Different letters in the same column indicate a significant difference among the samples (*p* < 0.05).

**Table 3:** Main thermal parameters of the thermo-compressed bio-based high-density polyethylene (bio-HDPE) films containing naringin (NAR), gallic acid (GA), caffeic acid (CA), and quercetin (QUER) in terms of melting temperature (T_m_), normalized melting enthalpy (ΔH_m_), degree of crystallinity (*X_C_*), onset oxidation temperature (OOT), and oxidation induction time (OIT).

Film	T_m_ (°C)	ΔH_m_ (J/g)	*X_C_* (%)	OOT (°C)	OIT (min)
bio-HDPE	134.9 ± 0.9 ^a^	187.8 ± 1.6 ^a^	64.1 ± 0.9 ^a^	223.7 ± 1.6 ^a^	4.5 ± 0.4 ^a^
bio-HDPE + NAR	132.1 ± 0.8 ^b^	186.9 ± 1.8 ^a^	63.8 ± 0.8 ^a^	232.2 ± 1.4 ^b^	9.2 ± 0.5 ^b^
bio-HDPE + GA	131.7 ± 0.7 ^b^	186.1 ± 1.9 ^a^	63.5 ± 1.0 ^a^	266.7 ± 1.9 ^c^	109.3 ± 1.6 ^c^
bio-HDPE + CA	134.1 ± 0.8 ^a^	171.3 ± 1.3 ^b^	58.5 ± 0.7 ^b^	253.6 ± 2.1 ^d^	42.7 ± 0.9 ^d^
bio-HDPE + QUER	131.8 ± 0.7 ^b^	187.1 ± 2.0 ^a^	63.9 ± 0.9 ^a^	265.2 ± 1.9 ^c^	137.9 ± 1.8 ^e^

^a–e^ Different letters in the same column indicate a significant difference among the samples (*p* < 0.05).

**Table 4:** Main thermal decomposition parameters of the thermo-compressed bio-based high-density polyethylene (bio-HDPE) films containing naringin (NAR), gallic acid (GA), caffeic acid (CA), and quercetin (QUER) in terms of onset degradation temperature measured for a mass loss of 5% (T_5%_), temperature of maximum degradation (T_deg_), and residual mass at 700 °C.

Film	T_5%_ (°C)	T_deg_ (°C)	Residual Mass (%)
bio-HDPE	355.1 ± 1.2 ^a^	473.7 ± 1.0 ^a^	0.30 ± 0.94 ^a^
bio-HDPE+NAR	376.1 ± 0.5 ^b^	481.6 ± 0.8 ^b^	0.27 ± 1.21 ^a^
bio-HDPE+GA	366.3 ± 0.8 ^c^	482.1 ± 1.1 ^c^	0.29 ± 1.16 ^a^
bio-HDPE+CA	371.7 ± 1.0 ^c^	479.4 ± 1.2 ^b^	0.25 ± 0.92 ^a^
bio-HDPE+QUER	362.2 ± 0.8 ^b^	481.3 ± 0.5 ^c^	0.28 ± 1.63 ^a^

^a–c^ Different letters in the same column indicate a significant difference among the samples (*p* < 0.05).

## Data Availability

Data is contained within the article.
